# “What's happening over there?”: a study of the current state of services, challenges, and prospects in Nigerian medical libraries

**DOI:** 10.5195/jmla.2020.607

**Published:** 2020-07-01

**Authors:** Biliamin Oladele Popoola, Ngozi Celestina Uzoagba, Nafisa Rabiu

**Affiliations:** 1 bpopoola@unimed.edu.ng, Systems and Evidence-Based Medicine Librarian, University Library, University of Medical Sciences, Ondo State, Nigeria; 2 ngozi.uzoagba@unn.edu.ng, Senior Librarian, Medical Library, College of Medicine, University of Nigeria, Nsukka, Nigeria; 3 rabiu.nf@unilorin.edu.ng, Assistant Lecturer, Department of Library and Information Science, University of Ilorin, Nigeria

## Abstract

**Objective:**

The authors examined the current state of service delivery, collections, and challenges in Nigerian medical libraries.

**Methods:**

We employed a descriptive mixed method research design using a cross-sectional quantitative survey of Nigerian medical librarians and qualitative interviews with heads of selected Nigerian medical libraries.

**Results:**

Respondents indicated that the US National Library of Medicine classification scheme is most commonly used to organize the resources of medical libraries in Nigeria. Respondents indicated that library users have a high understanding about the library but exhibit low usage of library services. Nigerian medical libraries have social media accounts but use them infrequently. Most medical librarians do not provide specialized services to health care professionals, and monographs are the major information resources in their collections. Most medical librarians in Nigeria have beginner-level knowledge of systematic reviews and evidence-based medicine and rarely organize training for library users.

**Conclusion:**

Our findings show that services offered by medical libraries in Nigeria are still evolving. Identified skill deficits among medical librarians need to be addressed. The country's library associations and international programs in developing countries should focus on providing continuing education and training of Nigerian medical librarians to enhance their support for medical education and practice in Nigeria.

**Figure:**
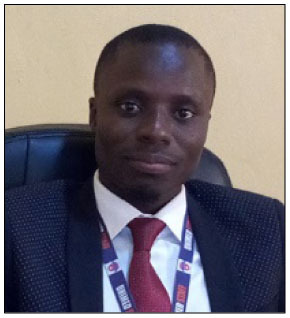
Biliamin Oladele Popoola

## INTRODUCTION

Medical librarianship is one of the oldest specializations in the field of library and information science, as evidenced by the formation of the US Medical Library Association (MLA) in 1898 [1], a time when there were few sectors in librarianship. Over its long existence, the rapidly changing field of medical librarianship has evolved to become an integral part of health care systems and medical education around the world [2]. However, the prominence and usefulness of the field have been more pronounced in the developed world, while medical libraries in developing nations grapple with survival and service delivery.

In Nigeria, a developing country in West Africa, the first medical library was established in 1910 at the Medical Research Institute Lagos [3], and until the early 1940s, this library and the Medical Headquarters Library Lagos were the only two medical libraries in the country [4]. These two libraries merged in 1945, combining their respective strengths in medical textbook and journal collections in one place, to form the Central Medical Library, Yaba Lagos [5], which is often inaccurately reported as the first medical library in Nigeria. By 1975, around seven medical libraries existed in the country [6], following the establishment of medical schools in Nigeria.

Following the modest beginnings of medical librarianship in Nigeria, the country now has at least 300 established medical libraries. Nigeria currently has 262 approved nursing schools [7], 51 medical and dental schools [8], and numerous health research institutes, ministries, and nonprofit organizations. All these institutions—by mandate or by necessity—have medical libraries. Some tertiary hospitals in the country also have medical libraries. Nevertheless, as there is no formal training for medical librarianship in the country, most services that are offered by these libraries are not specialized for health sciences clientele and are largely similar to those offered by general academic libraries.

Although medical librarianship continues to grow in Nigeria, the state, services, and challenges of medical libraries in the country were last reported on in the 1970s and 1980s [3, 6, 9, 10]. The latest of such reports, published in 1985, indicated that there were around twenty medical libraries and twenty-four medical librarians in the country [3]. It also stated that libraries were not considered a requisite part of medical education facilities in the country at that time, contradicting the current reality that the presence of libraries is required for medical school accreditation in Nigeria. Thus, there has been a gap in the literature about developments in the country's medical libraries, except for a recent report highlighting four trends in Nigerian medical librarianship: an increase in the utilization of information and communication technology for library operations, the inclusion of electronic resources in library collections, a shift to electronic means of searching for information, and the replacement of library orientations with information literacy training for users [11].

However, despite the outdated nature of most documented reports on the state of medical librarianship in Nigeria, some of the challenges that they identified still exist in the country's medical libraries. These challenges include a lack of adequate personnel, central libraries' control over medical libraries in universities, lack of proper library buildings, unavailability of a national library of medicine, need for cooperation among libraries, inadequate bibliographic control of medical literature, user communities' irregular use and nonuse of the library, and lack of formal training for medical librarians in the country [3, 6, 9, 12].

The aim of this study was to examine and update the literature on the state of service delivery in Nigerian medical libraries. The authors' specific objectives were to:

1. identify the classification schemes in use in Nigerian medical libraries;
2. examine Nigerian librarians' perceptions of users' understanding and usage of medical libraries;
3. determine whether Nigerian medical libraries use social media to communicate with users;
4. identify the availability of specialized services for health care professionals in Nigerian medical libraries;
5. describe the types of information resources most commonly held in Nigerian medical library collections;
6. determine Nigerian medical librarians' self-assessment of knowledge of evidence-based medicine (EBM) and systematic reviews (SR)/meta-analyses (MA);
7. ascertain whether Nigerian medical libraries organize training or seminars for users; and
8. highlight the challenges of and prospects for medical librarianship in Nigeria.

Being a typical developing country, updating the literature from a Nigerian perspective could inform the planning and design of medical library initiatives for international programs in Nigeria and other similar developing countries.

## METHODS

### Study design

We used a descriptive mixed methods research design to describe the present state of medical libraries and practice of medical librarianship in Nigeria. Following a survey consisting of open- and closed-ended questions, we performed semi-structured interviews to better understand the survey results related to the challenges and prospects of medical librarianship in Nigeria.

### Study participants and data collection

Our study population was librarians working in medical libraries in Nigeria. For the survey, we designed and deployed a questionnaire using Google Forms. A link to the survey was circulated among librarians using the Nigerian Library Association's email discussion list and the WhatsApp Group of the Medical Library Association of Nigeria (MLA-NG). The survey, designed based on reviewed literature, had four sections: demographic profile, library patronage and social media use, library services for users, and librarians' training and challenges.

We also conducted key informant interviews with heads of medical libraries in Nigeria's first-generation universities: University of Ibadan, Obafemi Awolowo University, Ahmadu Bello University, University of Lagos, and University of Nigeria, Nsuka. The interview time was prescheduled with library heads, and permission was given by all interviewees for the interviews to be recorded using a smartphone. We conducted interviews in the head librarians' offices at their institutions, and the average duration of interviews was four minutes, thirty-five seconds.

### Data analysis

Quantitative data obtained through Google Forms was exported into SPSS for descriptive analysis (i.e., response frequencies and percentages). Interviews were transcribed, and their content was analyzed using thematic content analysis, which yielded two major themes: challenges of and prospects for medical librarianship in Nigeria.

## RESULTS

A total of 58 librarians responded to the survey from medical libraries across the country. None of the responses were incomplete; thus, all responses were included in the analysis. Based on the 112 potential respondents on the MLA-NG WhatsApp Group at the time of conducting this study, our response rate was 52%.

### Survey results

#### Respondent demographics.

Most survey respondents were 41–50 years old (31%) or 51–60 years old (31%). Fewer were 31–40 years old (28%), ≥61 years old (7%), or 20–30 years old (3%), and none were <20 years old. Most respondents reported working in academic medical libraries (67%), with fewer working in special medical libraries (23%) or hospital libraries (10%). Respondents reported ≥10 years (40%), 7–9 years (33%), 1–3 years (17%), or 4–6 years (10%) of medical library experience. No respondents reported <1 year of experience in the field, corroborating our finding of few young professionals working in the field of medical librarianship in Nigeria. Most respondents (60%) had a master of library science (MLS) or master of library and information science (MLIS) as their highest degree. Some respondents had a doctorate (PhD) (26%) or bachelor's degree (14%) as their highest degrees.

Nigeria is divided into 6 geopolitical zones, with significant socioeconomic differences among zones. Most respondents were located in the South-West zone (54%), whereas fewer were in the North-West (16%), North-Central (14%), South-South (6%), South-East (5%), or North-East (5%) zones. The southern region of Nigeria is educationally more developed than the northern region and is home to 4 of the 5 first-generation universities in Nigeria. This difference was reflected in that 65% of respondents were from the southern region and 35% of respondents were from the northern region.

#### Classification schemes used in Nigerian medical libraries.

Despite being specialized libraries, medical libraries in Nigeria had differing standards for organizing resources. Most respondents reported using the US National Library of Medicine (NLM) classification scheme (43%), followed by the US Library of Congress (LC) classification scheme (32%), a combination of NLM and LC classification schemes (19%), Dewey Decimal Classification (4%), and Universal Decimal Classification (4%).

#### Nigerian librarians' perceptions of users' understanding and usage of medical libraries.

**Figure 1:**
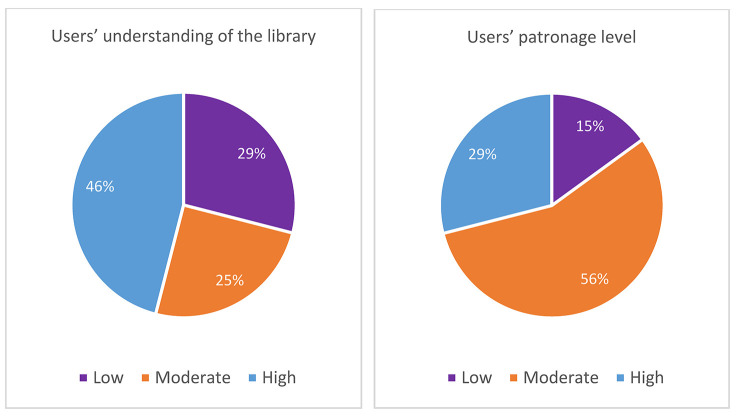
Librarians' perceptions of users' understanding and usage of Nigerian medical libraries

Respondents rated users' understanding and usage of their libraries' facility and services using a Likert scale of “very high,” “high,” “moderate,” “low,” or “very low,” reflecting their subjective perceptions. Most respondents believed that users had a high understanding of their libraries but exhibited only a moderate level of library usage (Figure 1).

#### Nigerian medical libraries' use of social media to communicate with users.

**Figure 2:**
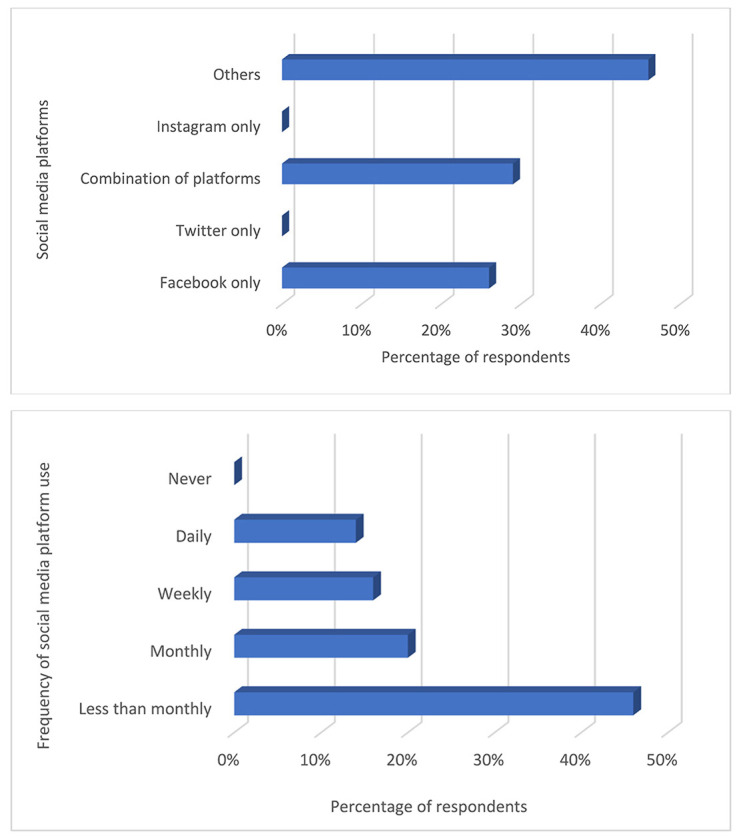
Social media accounts used to communicate with Nigerian medical library users (top) and frequency of social media use (bottom)

We investigated which social media platforms popular in Nigeria were used by medical libraries to communicate with their users. We found that most respondents had accounts on “other” (i.e., unspecified) social media platforms—aside from Facebook, Twitter, and Instagram—to communicate with library users (Figure 2). Other respondents described having library accounts on a combination of different social media platforms or only Facebook. However, most respondents reported rarely using these social media platforms to communicate with library users.

#### Availability of specialized services for health care professionals in Nigerian medical libraries.

An open-ended item in the survey was used to determine whether respondents provided any specialized services for health care professionals. We found that most respondents (53%) reported offering no specialized service for health care professionals. Some respondents offered selective dissemination of information, current awareness services, or reference services (31%) and literature searching (7%) to health care professionals. Notably, some respondents (5%) reported providing “health talk” services in their libraries, referring to organizing lectures on health topics for outreach to user communities. Also, some respondents (3%) offered “borrow-a-librarian” services, referring to the ability of health care professionals to request the service of an embedded librarian in their research or clinical practice for a period of time.

#### Major types of information resources held in Nigerian medical library collections.

**Figure 3:**
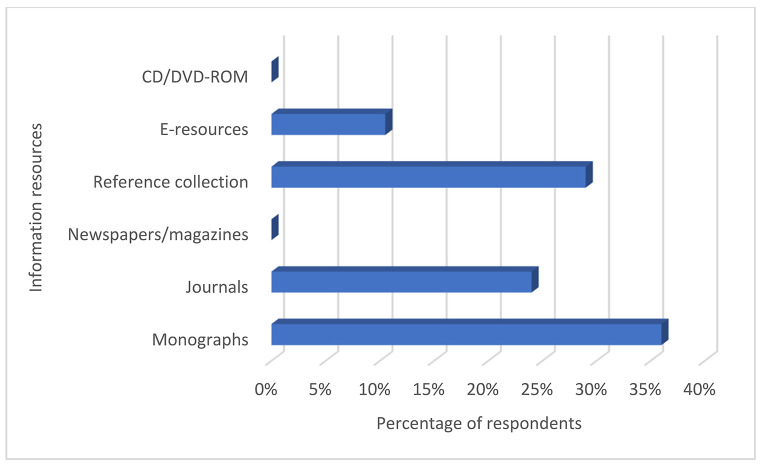
Major information resources in Nigerian medical library collections

Most respondents reported that monographs constituted most of their libraries' collections (Figure 3). Others reported journals and reference resources as the bulk of their collection, whereas a minority reported electronic resources as constituting most of their libraries' collections.

#### Nigerian medical librarians' self-assessment of their knowledge of evidence-based medicine (EBM) and systematic reviews (SR)/meta-analyses (MA).

**Figure 4:**
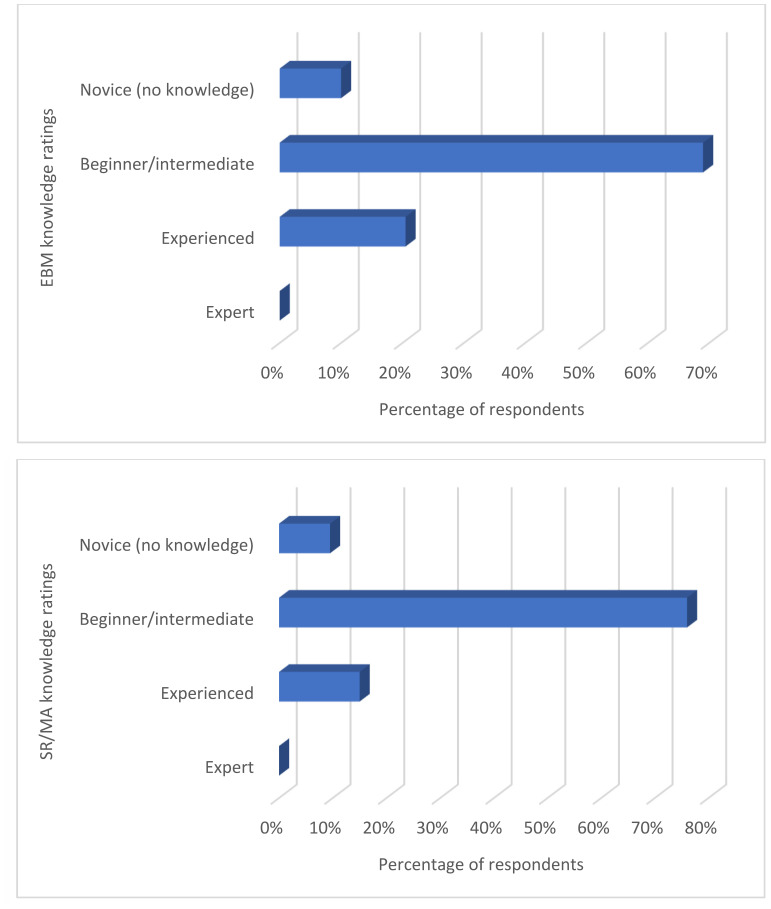
Nigerian medical librarians' knowledge of evidence-based medicine (EBM) (top) and systematic reviews (SR)/meta-analyses (MA) (bottom)

Respondents were asked to rate their knowledge of EBM and SR/MA using Likert scales. Most respondents rated themselves as being at a beginner or intermediate knowledge level for both topics (Figure 4).

#### Nigerian medical libraries' organization of training or seminars for users.

Half of respondents (50%) reported that their library had not organized any training or seminars for users in the previous 6 months. However, 41% and 9% of respondents reported that their libraries organized 1–2 or 3–4 trainings or seminars in the previous 6 months, respectively.

#### Challenges of medical librarianship in Nigeria.

Half of respondents (50%) agreed that an inability to access electronic information resources was a significant barrier to improved services in Nigerian medical libraries. Over half of respondents also agreed that a lack of training for medical librarians in Nigeria (53%) and low usage of libraries (57%) were challenges to their service delivery. Slightly less than half of respondents (48%) agreed that inadequate library space was a challenge.

### Interview results

We interviewed the heads of medical libraries in the five first-generation universities in Nigeria to reinforce the survey data regarding the challenges of medical librarianship in Nigeria. Of the five interviewees, three had eight to ten years of experience, one had three years of experience, and one had more than twenty years of experience in medical librarianship. We used a thematic content analysis approach to analyze the interview data and arrived at two major themes: challenges of and prospects for medical librarianship in Nigeria.

#### Challenges confronting medical librarianship in Nigeria.

A major challenge confronting medical librarianship in Nigeria was the lack of interlibrary cooperation, which was noted by all five interviewees as a problem that hampered the delivery of literature searching services in their libraries. Another challenge was that medical libraries' budgets, staff recruitment, and administration were managed by the central library systems of universities. Other challenges included inefficient Internet availability; lack of professional recognition of librarians; inability to obtain resources and books for the library, “especially…local journals…published across the country”; delays in journal delivery; and lack of formal training for librarians working in medical libraries.

#### Prospects for medical librarianship in Nigeria.

When asked what the prospects for health sciences librarianship in Nigeria were and if they would advise young Nigerian librarians to take jobs in medical libraries, all interviewees affirmed that the field of medical librarianship had high prospects in the country. One interviewee considered the field to be “very important, and medical librarians are part of the needed professionals” in the health care system. However, two interviewees cautioned that the projected bright prospects for the field of medical librarianship in Nigeria depended on the activities of the librarians and whether “people in…leadership are up and doing.”

## DISCUSSION

The aim of this study was to examine the current state of service delivery in Nigerian medical libraries. We found that most medical librarians in the country were middle-aged (i.e., forty-one to sixty years old). This might reflect inactive recruitment or lack of attraction of younger librarians to the field of medical librarianship in Nigeria, despite that the technological savviness of younger professionals could enhance medical library service delivery. We also found that the US NLM classification scheme was most commonly used to organize medical library resources, which contrasted with previous reports of the use of the Dewey Decimal Classification scheme in two developing countries [13, 14]. This finding could be valuable to new medical libraries staffed by individuals with no previous experience or training in medical librarianship, and the variation in responses showed a need for wide consultation by new libraries to ascertain the most suitable classification scheme for their needs.

Most medical librarians did not report high usage of their libraries, suggesting that medical libraries in Nigeria must take active steps toward encouraging the use of their facilities and services. Low usage could be a result of users' enhanced skillfulness with information technology, which might even surpass that of librarians in some cases. Library usage could be assured if users are not able to circumvent the library to obtain library resources and services elsewhere. Planning for resource and service provision together with users, rather than on behalf of users, could accentuate the benefits of library use to potential users. Such benefits might arise from improving library aesthetics, creating functional and versatile spaces, taking strategic approaches to service delivery, employing problem-solving skills, and focusing on interpersonal and user-centered relationships. Similar reports on library users' patronage of Nigerian medical libraries corroborated the need for libraries to innovatively address the low usage of their facilities and services [2, 15, 16].

Similar to an earlier report [17], we found that most medical libraries in Nigeria had accounts on social media platforms to communicate with users but rarely used these platforms. We found that libraries mostly had accounts on “other” (i.e., unspecified) social media platforms. In a Nigerian context, this could largely refer to the use of WhatsApp or similar instant messaging platforms. Consistent with these findings, a previous study reported high awareness of the use of social media for service delivery in Nigerian libraries [18], but another study reported that the use of social media platforms to deliver services to Nigerian library users was low [19]. Nigerian medical libraries could leverage the reported importance of social media for library services [20, 21] by using the platforms to engage with users to improve library patronage. That is, social media could bridge some physical and service awareness gaps that exist between the library and its users.

Service delivery is the core duty of libraries and must be actualized to meet the needs of all library user groups. We found that most medical libraries in Nigeria did not offer any specialized services to health care practitioners. However, modern medical libraries should offer services that lead to including librarians as part of health care teams. These types of service models are exemplified in non-Nigerian settings in developed countries, such as the Welch Medical Library's Clinical and Evidence Based Practice program at Johns Hopkins University and the Informationist Program at the National Institutes of Health Library.

SR/MA services have also become popular in medical libraries in developed countries. However, we found that most medical librarians in Nigeria were at the beginning stage of SR/MA knowledge. This beginning stage explained why no respondents reported offering support for SR/MA, which are important components of medical education and practice today. Brettle described the need for librarians to acquire SR skills [22], and many medical librarians have played roles in medical schools and clinics as clinical, embedded, or SR librarians [23, 24]; therefore, the challenge of attaining SR/MA skills should be addressed seriously if librarians are to attain relevance in the health sector. Some previous studies also identified the need to address this knowledge gap among medical librarians [25–27], especially now that demand for medical library services in EBM and SR/MA is on the rise globally [28, 29].

This study further revealed that medical libraries in Nigeria had monographs as the major part of their collections. This agreed with an earlier report that monographs and journal back runs were the most available resources in medical libraries in Nigeria [30]. This finding could coincide with the underutilization of available electronic information resources—such as HINARI, JStor, and Cochrane Library—which offer free or cheap access to expansive electronic resources for developing countries. Aside from this, medical libraries could raise their reputation and gain relevance by organizing regular trainings, workshops, and seminars for their users, which would help address our finding that training for users was not frequently provided in Nigeria's medical libraries. Such training, workshops, or seminars would enable medical librarians to address the information literacy needs of health professionals and students [31, 32]. Focusing on electronic information resources and user training would considerably improve collection usage and service delivery in Nigeria's medical libraries. Medical libraries in developed nations, such as the United States, have already been witnessing a decline in print resources and repurposing their space for users [33].

Our findings confirmed known challenges that were previously described in the literature [9, 34] and revealed new challenges in current medical library settings in Nigeria. Many previously identified challenges might persist due to librarians' self-reported slowness to innovate and upgrade services and hesitance in demonstrating library value to their user communities. Medical librarians in Nigeria will have to deploy ingenuity in their service delivery to gain recognition and enhance patronage. Once the library is recognized as valuable and resourceful, institutional support and patronage could be assured to a greater extent. Furthermore, a vibrant library association could be key to addressing other professional challenges [11]. As our findings suggested a bright prospect for medical librarianship in Nigeria, medical librarians in the country only need to enhance their skills and services to overcome future challenges.

In light of our results, professional organizations have an opportunity to target learning toward areas of particular need to Nigerian medical libraries and librarians. The MLA-NG and Nigerian chapter of the Association for Health Information and Libraries in Africa could develop and organize focused skill acquisition trainings. These library organizations can also partner with larger associations that have similar goals, such as MLA and the European Association for Health Information and Libraries, which currently offer continuing professional development opportunities for librarians. Nigerian librarians can publicize the pursuit of these opportunities, many of which are offered online.

Medical librarianship in Nigeria is still evolving. Nigeria's medical libraries could identify opportunities to improve use of their collections by focusing on access to electronic information resources and offering more user-centered services. To remedy financial constraints, interlibrary collaborations for consortial subscriptions to electronic resources could be pursued. Furthermore, librarians' resourcefulness and participation in programs focused on their professional development in developing countries could improve the state of medical librarianship in Nigeria and globally. Despite the challenges described in this study, the future prospects for medical librarianship in Nigeria remain bright, if stakeholders can innovatively work together to enhance service delivery.

## SUPPLEMENTAL FILES

**Appendix A:** Questionnaire on health sciences librarianship in Nigeria

**Appendix B:** Qualitative instrument
