# Supplementary material for: “What's happening over there?”: a study of the current state of services, challenges, and prospects in Nigerian medical libraries
Source: J Med Libr Assoc. 2020 Jul 1;108(3):398–407. doi: 10.5195/jmla.2020.607 (PMC7441899; doi:10.5195/jmla.2020.607)
Supplement: Supplementary file 1 — Appendix A: Questionnaire on health sciences librarianship in Nigeria [file jmla-108-3-398-s01.pdf]

JMLA Journal of the Medical Library Association

SC
Supplemental content to
J Med Libr Assoc. Jul;108(3):dx.doi.org/10.5195/jmla.2020.607
www.jmla.mlanet.org
© Popoola, Uzoagba, Rabiu 2020

"What's happening over there?": a study of the current state of services, challenges, and prospects in Nigerian medical libraries

Biliamin Oladele Popoola; Ngozi Celestina Uzoagba; Nafisa Rabiu

APPENDIX A

Questionnaire on health sciences librarianship in Nigeria

(Deployed via Google Form at https://forms.gle/LiYhtg31gLBdtehQ7)

This questionnaire was designed to assess some aspects of health sciences librarianship in Nigeria. Kindly fill in your responses to the questions as most appropriate. Please click the "NEXT" button to flip through the pages. Thank you, B.O. Popoola (researcher)

Section A: Demographic profile

1. Age group:

Below 20 years ☐
20–30 years ☐
31–40 years ☐
41–50 years ☐
51–60 years ☐
Above 60 years ☐

2. What type of library do you work in?

Hospital library ☐
Medical library (academic) ☐
Medical library (special) ☐

3. How long (in years) have you worked in a health sciences library?

Less than 1 year ☐
1–3 years ☐
4–6 years ☐
7–9 years ☐
10 years and above ☐

4. Gender:

Male ☐
Female ☐

5. Highest qualification obtained:

Bachelor's of library science/bachelor's of library and information science (BLS/BLIS) ☐
Master's of library science/master's of library and information science MLS/MLIS ☐
Doctoral degree (PhD) ☐

JMLA Journal of the Medical Library Association

SC
Supplemental content to
J Med Libr Assoc. Jul;108(3):dx.doi.org/10.5195/jmla.2020.607
www.jmla.mlanet.org
© Popoola, Uzoagba, Rabiu 2020

6. In which geopolitical zone of Nigeria is your library located?

South-South ☐
South-East ☐
South-West ☐
North-West ☐
North-East ☐
North Central ☐

7. Which classification scheme is your library using to arrange its collection? (check all that apply)

Library of Congress Classification (LC) ☐
National Library of Medicine Classification (NLM) ☐
Dewey Decimal Classification (DDC) ☐
Universal Decimal Classification (UDC) ☐

Section B: Library patronage/social media use

8. How would you rate the level of users' patronage for your library and its services?

Very low ☐
Low ☐
Moderate ☐
High ☐
Very high ☐

9. How would you rate users' understanding of the importance of the library and what it stands for?

Very low ☐
Low ☐
Moderate ☐
High ☐
Very high ☐

10. Which of the following social media platforms does your library use to communicate with users? (check all that apply)

Facebook ☐
Twitter ☐
Instagram ☐
Others ☐

11. How often does your library update contents or information on the social media platforms chosen above?

Rarely ☐
Monthly ☐
Weekly ☐
Daily (every working day) ☐
Never ☐

Section C: Library services for users

12. Does your library offer any specialized service for health care practitioners? If yes, what name is the service called? (leave blank, if No) ___________________________

SC
Supplemental content to
J Med Libr Assoc. Jul;108(3):dx.doi.org/10.5195/jmla.2020.607
www.jmla.mlanet.org
© Popoola, Uzoagba, Rabiu 2020

13. How would you rate the level of your knowledge in evidence-based medicine practice?

Novice (no knowledge) ☐
Beginner ☐
Intermediate ☐
Experienced ☐
Expert ☐

14. How would you rate the level of your knowledge in systematic reviews/meta-analysis?

Novice (no knowledge) ☐
Beginner ☐
Intermediate ☐
Experienced ☐
Expert ☐

15. Which of the following resources does your library holds as its largest collection?

Monographs ☐
Journals ☐
Newspapers/magazines ☐
CD/DVD-ROM ☐
Reference collection ☐
E-resources ☐

16. In the past six months, how many trainings or seminars has your library organized for users?

None ☐
1–2 ☐
3–4 ☐
5 and above ☐

Section D: Librarians training and challenges

17. Have you attended or undergone any specialized training in health sciences librarianship in the past?

No ☐
Yes ☐
Not sure ☐

18. Which of the following do you agree is a barrier to your service delivery as a health sciences librarian? (check all that you agree with)

Inability to access electronic information resources ☐
Lack of training for medical librarians ☐
Low patronage from users ☐
Inadequate library space ☐
