# Supplementary material for: “What's happening over there?”: a study of the current state of services, challenges, and prospects in Nigerian medical libraries
Source: J Med Libr Assoc. 2020 Jul 1;108(3):398–407. doi: 10.5195/jmla.2020.607 (PMC7441899; doi:10.5195/jmla.2020.607)
Supplement: Supplementary file 2 — Appendix B: Qualitative instrument [file jmla-108-3-398-s02.pdf]

Journal of the Medical Library Association

Supplemental content to
J Med Libr Assoc. Jul;108(3):dx.doi.org/10.5195/jmla.2020.607
www.jmla.mlanet.org
© Popoola, Uzoagba, Rabiu 2020

"What's happening over there?": a study of the current state of services, challenges, and prospects in Nigerian medical libraries

Biliamin Oladele Popoola; Ngozi Celestina Uzoagba; Nafisa Rabiu

APPENDIX B
Qualitative instrument

Interview schedule

Interview date:

Interview time:

Institution:

Interviewee's gender: Male Female

[Prelude:] seek permission from interviewee to make an audio recording of the interview.

Section A: Demography

1. How long have you worked as a health sciences librarian?

Section B: Challenges

1. What are the challenges presently facing health sciences librarianship in the country?

[Probe:] If not mentioned, ask the interviewee if they think the following are part of the challenges:

a. Lack of standard for the practice of health sciences librarianship
b. Inexistence of interlibrary cooperation among health sciences libraries
c. Unavailability of formal training in medical librarianship
d. Shortage of professional staff in health science libraries

Section C: Prospects

1. What can you say about the prospects of health sciences librarianship in Nigeria?
2. Would you advise young Nigerian librarians to consider a career in health sciences librarianship?

Section D: Finally

1. Is there anything you would like to add to the discussion?
